# Lab-on-Chip for Exosomes and Microvesicles Detection and Characterization

**DOI:** 10.3390/s18103175

**Published:** 2018-09-20

**Authors:** Maria Serena Chiriacò, Monica Bianco, Annamaria Nigro, Elisabetta Primiceri, Francesco Ferrara, Alessandro Romano, Angelo Quattrini, Roberto Furlan, Valentina Arima, Giuseppe Maruccio

**Affiliations:** 1 CNR NANOTEC Institute of Nanotechnology, via Monteroni, 73100 Lecce, Italy; monica.bianco@nanotec.cnr.it (M.B.); elisabetta.primiceri@nanotec.cnr.it (E.P.); francesco.ferrara@st.com (F.F.); valentina.arima@nanotec.cnr.it (V.A.); giuseppe.maruccio@unisalento.it (G.M.); 2 Institute of Experimental Neurology, Division of Neuroscience, San Raffaele Scientific Institute, 20132 Milan, Italy; nigro.annamaria@hsr.it (A.N.); romano.alessandro@hsr.it (A.R.); quattrini.angelo@hsr.it (A.Q.); furlan.roberto@hsr.it (R.F.); 3 STMicroelectronics, Via Monteroni, I-73100 Lecce, Italy; 4 Department of Mathematics and Physics, University of Salento, via Monteroni, 73100 Lecce, Italy

**Keywords:** lab-on-chip, microfabrication, microfluidics, biosensors, extracellular vesicles, exosomes

## Abstract

Interest in extracellular vesicles and in particular microvesicles and exosomes, which are constitutively produced by cells, is on the rise for their huge potential as biomarkers in a high number of disorders and pathologies as they are considered as carriers of information among cells, as well as being responsible for the spreading of diseases. Current methods of analysis of microvesicles and exosomes do not fulfill the requirements for their in-depth investigation and the complete exploitation of their diagnostic and prognostic value. Lab-on-chip methods have the potential and capabilities to bridge this gap and the technology is mature enough to provide all the necessary steps for a completely automated analysis of extracellular vesicles in body fluids. In this paper we provide an overview of the biological role of extracellular vesicles, standard biochemical methods of analysis and their limits, and a survey of lab-on-chip methods that are able to meet the needs of a deeper exploitation of these biological entities to drive their use in common clinical practice.

## 1. Introduction

The extracellular vesicles (EVs) identify a group of membrane enclosed structures that are released from cells in a constitutive or induced manner. Typically, EV subpopulations are divided on the basis of their dimensions into specific categories including exosomes (~100 nm in diameter), microvesicles (MVs, 100–1000 nm), and apoptotic bodies (1–5 μm) [1], which differ in several important parameters such as nucleic acid content, density of membrane lipid packing, protein to lipid ratio and morphology [2]. They are considered both as a snapshot of the cells from which they originate and as depository of some important information and they are becoming more and more of interest for their role as intercellular messengers, also including the possibility of playing crucial roles in the spreading of diseases. For these reasons, increasing attention is currently devoted to EVs for their potentiality as novel biomarkers, prognostic tools, as well as therapeutic targets [3]. Their consideration as disease’s molecular landscape is a prominent research topic in precision medicine. Moreover, the presence of EVs in biofluid samples (blood, urine, saliva, cerebrospinal fluid) is a great opportunity for diagnostic innovation as they can be potential candidates for the so-called “liquid biopsy”, which is emerging as a powerful method to monitor treatment efficacy, drug resistance, and evolution of disease, avoiding the need for repeated invasive examinations on patients over time [4].

Despite the promising diagnostic value of EVs, including in particular exosomes, their purification and characterization are technically challenging and standard methods of analysis are not able to take advantage of their potentiality. In order to overcome these limitations, the development of integrated and inexpensive point-of-care (POC) devices can cover the unmet needs, leading to a deeper exploitation and many tools have been developed in the last years to improve EVs sorting efficiency, to ease their separation, and to accelerate analysis processes in compact devices. The obvious advantages of microfluidics, in any case, pass through the narrow bottleneck of integration and automation, in addition to mass production and translation in clinical practice, which is still a barrier toward a higher degree of innovation and industrial exploitation for POC systems. 

In this article, after an overview concerning the biological relevance of EVs and performances and limits of current standard methods of analysis, we review the state-of-the-art of on-chip analysis modules for MVs and exosomes, with a focus on isolation tools (passive and active) from body fluids and the possibility for on-chip counting of extracellular vesicles, and to investigate their proteins and nucleic acids content after the induced lysis of membranes. Considerations are also provided concerning integration needs to push these technologies to be available in clinical practice. 

## 2. Exosomes and Microvesicles from a Biological Point of View

### 2.1. Extracellular Vesicles: Overview and Biogenesis

EVs are small cell-derived vesicles that are released into the extracellular space by most cell types [5]. They have been found and isolated from diverse body fluids, including blood, urine, saliva, mother milk, amniotic fluid, semen, cerebrospinal, and synovial fluids [6,7]. EVs have emerged as an additional mechanism of cell-cell communication due to their ability to carry, transfer and exchange signaling molecules, proteins, lipids, and nucleic acids (including mRNA, miRNA, and DNA) [8,9,10,11,12]. Two main categories of EVs are secreted from cells, exosomes, and microvesicles (MVs), differing for sizes and biogenesis mechanisms. In addition to these two classes, apoptotic bodies are large (1000 to 5000 nm in diameter) EVs that are specifically released by cells undergoing apoptosis [13,14,15].

**Figure 1:**
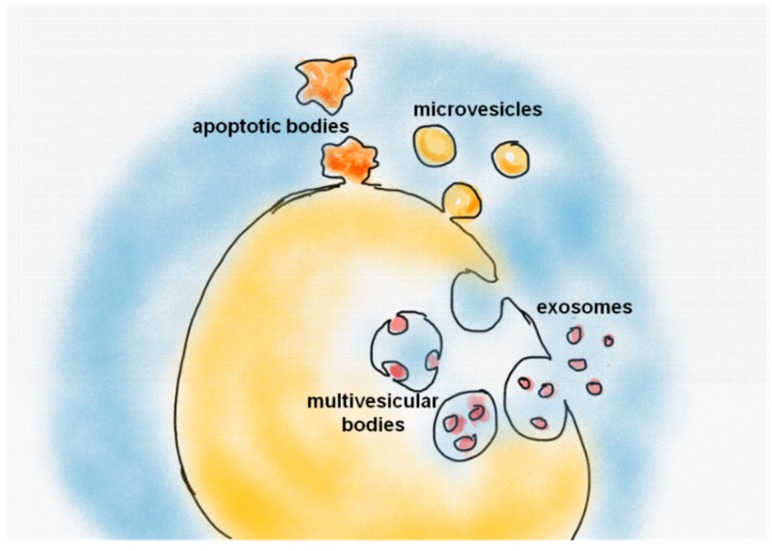
Schematic representation of the different type and biogenesis of extracellular vesicles. Figure not in real proportions scale.

Exosomes, which are the most studied EVs, constitute a rather homogeneous population of small spherical vesicles of approximately 30 to 100 nm in diameter. Exosomes originate by the inward budding of endosomal membranes to form multivesicular bodies (MVBs) and are released in the extracellular environment by MVB fusion with the plasma membrane [8,16]. On the other hand, MVs are a heterogeneous population of large vesicles ranging from 100–1000 nm in diameter that originate from the plasma membrane of the cells by direct outward budding and shedding [17,18] (Figure 1). 

The EV composition closely reflects their biogenesis (exosomes or MVs) and the physiological and environmental conditions of the cell of origin, including substantial portions of cytosol and membrane-associated material [19]. Exosomal membranes are enriched in elements of lipid rafts (e.g., GM1 gangliosides and transferrin receptors [20]; cholesterol, ceramide, and sphingomyelin [21]). Moreover, they contain several endosome-specific proteins, including Tsg101, Alix, and the tetraspanins CD9, CD63, and CD81, which are often used to differentiate them from other EV populations. MV release depends on calcium influx, scramblase, floppase, cytoskeleton reorganization, and plasma membrane phospholipid re-distribution [5,22]. In particular, MVs expose phosphatidylserine (PS) on the outer leaflet of the membrane and they can typically be identified by flow cytometric analysis using Annexin-V staining [23]. Moreover, they are enriched in cholesterol, sphingomyelin, and ceramide. Conversely to exosomes, few MV specific markers have been identified. However, CD40 ligand, ADP-ribosylation factor 6 (ARF6), and different protein associated to lipid rafts, such as integrins and flotillins, are reported as MV markers [24,25,26,27]. Of note, the fact that exosomes and MVs sharing different markers are commonly found in the same extracellular fluids and exert similar biological effects [28], makes the currently available purification methods inefficient to definitely isolate and discriminate the two types of vesicles.

### 2.2. The Physiological and Pathological Role of EVs

In cells and organisms, EVs (exosomes and MVs) support two major biological needs, the removal of misfolded proteins and superfluous molecular material from the cell and the transfer of specific biomolecules between cells both locally and over long distance through biological fluids (e.g., blood) [8,29,30]. With regard to the last aspect, EVs can deliver their content to the recipient cells by fusion with plasma membrane or endocytosis and thus modulate the molecular pattern and the physiology of target cells [31]. In this context, exosomes and MVs play an important role not only in intercellular communication, but also in physiological processes, such as coagulation, angiogenesis, cell survival, tissue repair, stem cell maintenance, modulation of immune responses, and inflammation [26,32,33,34,35,36,37,38,39,40,41,42,43,44]. Moreover, even though EVs are constitutively released in physiological conditions, their release can increase following cellular activation [45], as well as in many pathologic conditions such as cancer, cardiovascular complication, inflammatory, and neurodegenerative disorders. In this respect, EV count in biological fluids may be indicative of an ongoing aberrant process and it can be used as potential predictive and diagnostic biomarkers for a large number of pathologies [46].

A large body of evidence suggests that cancer cells release higher amounts of EVs, which should be involved in processes leading to transformation from non-malignant to malignant phenotypes in the recipient cells [47,48,49,50,51,52]. Recent studies reveal that EVs released by cancer cells can affect tumor microenvironment inducing stromal cells to adopt proangiogenic, prometastatic, or immune suppressive phenotype [53]. Moreover, cancer EVs can contribute to cancer progression remodeling the extracellular matrix (ECM) by delivering growth factors, adhesion molecules, and metalloproteases [54,55]. Recently, EVs have been reported to participate in the pre-metastatic niche formation altering the behavior of bone marrow-derived progenitors or resident specialized cells [56]. 

In cardiovascular physiopathology, EVs show a Janus-faced behavior in cardiovascular physiopathology [57,58], working as positive regulators of vascular homeostasis and endothelial regeneration [59] and conversely contributing to atherosclerosis development and progression [60]. 

Both exosomes and MVs are found to be involved in neurodegenerative and neuroinflammatory disorders [42,61] where they may exert a neuroprotective action (supporting the removal of toxic proteins and the transfer of neuro-protective molecules) or a neurotoxic role (promoting the transfer of the toxic misfolded proteins to the target neural cells). Actually, exosomes are linked to both Alzheimer and Creutzfeldt-Jakob diseases [62] as vehicles/vectors of the β-amyloid peptide [63,64] and prion protein [65]. In general, microglia EVs have been shown to work as vehicle of a number of pathogenic proteins, i.e., the pro-inflammatory cytokine IL-1β [66], pathogenic β-amyloid, and tau protein [67,68], which are toxic to target neurons. The detrimental role of EVs in neurodegenerative and neuroinflammatory is also recently supported by data showing the increase of myeloid-derived MVs in the cerebrospinal fluid (CSF) of patients suffering with Alzheimer’ s disease [68,69] and severe forms of multiple sclerosis [70]. However, the lack of sensitive preparatory and analytical technologies for their detection and isolation poses a barrier to clinical translation.

### 2.3. Standard Methods for EVs Isolation

Extracellular vesicles constitute an ideal tool to obtain information on cells residing in inaccessible organism sites (such as the brain) and represent a potential clinical biomarker to provide information on the pathophysiology of several diseases. However, the lack of standardized and sensitive methods and technologies for their isolation, enrichment, and detection from biological fluids poses a serious barrier to biomedical investigation and clinical translation.

Currently, EVs are isolated by a variety of methods that influence amount, type, and purity of the recovered EVs [71,72]. Each isolation technique presents advantages and disadvantages and the choice of methods should be based on different factors, such as starting material, volume, desired grade of purity, and isolation purpose (research, therapeutic, or diagnostic use). Table 1 enlists the most widely used EV isolation strategy. Differential centrifugation is the most commonly used method to isolate EVs [73,74,75]. The EVs are separated on the basis of their density by consecutive centrifugation steps. The protocol consists in a brief centrifugation step at low centrifugal force (300 g) to discard cells and debris and subsequent centrifugation steps at higher speeds to isolate EVs populations based on their different density (i.e., 10,000 g for MVs and 100,000 g for exosomes). The method was firstly described by Raposo and colleagues, and then widely applied with slight variations to isolate EVs from biological fluids and conditioned media [72,73,76]. Despite that isolation is of widespread use, differential centrifugation shows several drawbacks, including vesicle aggregation, protein and soluble factors contamination, inefficient isolation/recovery, and lengthy and laborious processing [77]. Therefore, this approach is often combined with density gradient centrifugation to improve EV recovery and purity. Recently, iodixanol-based gradient (OptiPrepTM) was adopted to efficiently separate and characterize different subtypes of EVs [75,78,79,80]. 

Polymer-based precipitation methods are also widely used for EV isolation. The method relies on precipitation reagents, such as polyethylene glycol, to reduce EV solubility and isolate EVs from a variety of biological fluids. This technique achieves higher yields of EVs than differential centrifugation [81,82] and several companies have introduced EV isolation kits based on this method (e.g., ExoQuick, Exo-Spin). Recently, Shin and colleagues optimized the method coupling polyethylene glycol and dextran aqueous two-phase system (ATPS) to efficiently isolate EVs from human body fluids [83]. However, this method is expensive for large-scale application, requires long incubation times and results in a low purity EV preparation due to co-precipitation of other contaminants. 

Size-exclusion chromatography (SEC) separates EVs of different sizes by filtration through columns containing spherical beads with pores of a specific size distribution [84]. Separation occurs because the small molecules/vesicles are delayed when entering into the pores, while large molecules/vesicles are excluded from the pores and they pass directly through the column. Several factors can influence the efficiency and resolution of the separation, including pore size, column dimension, column packing, mobile-phase composition, and flow rate. SEC has been recently applied to isolate EVs from complex biological fluids, such as plasma, urine and milk [85,86,87,88,89,90], and studies comparing SEC to differential centrifugation suggested that SEC provides higher EV recovery and purification [91].

Immunoaffinity capture-based techniques employ magnetic beads conjugated with specific antibodies directed against proteins expressed on EV surface to recognize and isolate EVs [92,93,94]. This method selects specific subpopulations of EVs without considering their size or density [84], and they can be coupled to flow cytometry, western blotting, and real-time PCR to further characterize EVs. Immunoaffinity isolation is consistently used for isolation of EVs from complex biological fluids and/or small volumes [75]. Recently, an optimized ELISA assay has been adopted for the capture and quantification of EVs from serum and plasma [95].

### 2.4. Standard Methods for EVs Characterization

Currently, several techniques are employed to characterize EVs, including electron microscopy, atomic force microscopy (AFM), dynamic light scattering (DLS), nanoparticle tracking analysis (NTA), tunable resistive pulse sensing (TRPS), flow cytometry, enzyme linked immune-sorbent assays (ELISA), and western blotting (WB). For a summary of these techniques, along with advantages and limitations, see Table 2. These methods are widely used to measure the physical (e.g., size, morphology, concentration) and molecular/biological (e.g., protein content, surface marker expression) features of EVs. Due to the small differences in the physical properties and composition of the different EV subpopulations the combination of diverse techniques is required for qualitative and quantitative EVs characterization, and the International Society of Extracellular Vesicles (ISEV) recommends to use at least two independent technologies to characterize individual EVs [71].

Electron microscopy is a valuable tool to assess the size and morphology of EVs, to confirm purity of EVs preparation, and, in combination with immunogold labeling (in which colloidal gold conjugated with antibodies is used for staining), to identify protein located in EVs [93]. AFM also provides information on the size distribution and morphology of EVs; moreover, AFM is a suitable technique to obtain details on the mechanical properties of EVs surface. However, both microscopy techniques cannot be employed to measure the concentration of vesicles [96]. 

DLS and NTA methods rely on Brownian motion of particles to measure size distribution and concentration of EVs [97,98,99]. DLS measures the intensity fluctuations of the scattered light resulting from the Brownian movements of the particles when the sample is illuminated with a light beam. NTA measures Brownian movements on a particle-by-particle basis tracking movements by image analysis. Both techniques provide accurate size information of monodisperse samples containing EVs that are of a specific size but are less accurate and should be considered with caution when heterogeneous EV samples are analyzed. 

The TRPS method, which is based on the Coulter principle, can accurately determine the size, charge, and concentration of EVs [100,101,102]. The physical properties of vesicles are measured on a particle-by-particle basis detecting the changes in the ionic current occurring when they pass through a size-tunable nanopore.

Flow cytometry is the most commonly used method for EVs analysis. This technique is frequently available in biological laboratories and allows for rapidly analyzing thousands of EVs in a single sample and a large number of samples [103]. Moreover, by using specific fluorochrome-conjugated antibodies, flow cytometry is ideal to determine the expression of specific EVs markers and classify/quantify the vesicle populations accordingly. However, the size detection limit of conventional flow cytometry is approximately 300 and the detection of smaller EVs (exosomes) is presently challenging. Nevertheless, high-sensitivity flow cytometer technologies have been recently developed that are able to detect particles as small as 100 nm in diameter [104,105]. ELISA and western-blot are classical immunological methods that provide information on the presence/expression of proteins and surface markers in EVs and could be very useful as indirect methods for quantifying and characterizing proteins in purified vesicle preparations [106,107]. 

Recently, the use of special techniques, such as Raman microspectroscopy, micro nuclear magnetic resonance, and small-angle X-ray scattering (SAXS) has been explored as alternative methods to study EVs [108,109,110]. These methods revealed great potential to gain useful information on the physical and molecular properties of EVs.

## 3. Isolation of Exosomes and Microvesicles from Biological Fluids through Microfluidics

### 3.1. The Rise of Lab-on-Chip for Exosome and Microvesicles Analysis

Exosome isolation is an important target of current research, being the first step towards the analysis of selected exosome sub-populations whose external markers, size, and number can be correlated to physiological or pathological conditions. To exploit circulatory exosomes or MVs as novel biomarkers, large scale, rapid, high-throughput, and reproducible methods for their isolation, detection, and characterization are required. Lab-on-chip (LOC) technologies offers customizable opportunities for their separation/isolation [111,112,113] on the basis of various physicochemical parameters, such as immunochemistry [114,115], sieving, acoustic wave [116], and field flow fractionation [117] to obtain micro- and nano-scale devices exhibiting high accuracy, precise control, lower energy consumption, and minimal sample size. 

Multifunctional LOCs are realized by microfluidic technologies using high level microfabrication techniques traditionally developed for mass production in micro- and nanoelectronics field (photolithography, MEMS technologies, etching, bonding) [118]. This approach uses rigid materials, like silicon, glass, quartz, metals, and some organic polymers. Among them, glass is more suitable for LOC applications because of its optical transparency, its surface stability [119] and its easy manufacturing [120,121]. Beyond glass, polymers like SU-8 are interesting for their chemical and mechanical properties, their low cost, and ease of fabrication [122,123,124]. 

However, although providing robust chips, stiff materials are not always advantageous for biological applications; therefore, elastomers are preferred, such as polydimethylsiloxane (PDMS), which is an optically transparent, non-toxic, and inexpensive material manufactured by soft lithography [125]. To match the requirements of specific applications, the surface of PDMS is often modified by plasma treatments or surface grafting [126], which introduce the desired functionalities [127] and reduce the undesired adsorption of chemicals and biomolecules [128,129,130]. The most part of chips described in the next sections and used for exosomes sorting/detection are made of PDMS and are produced as described here.

Microfluidic technologies allow for producing LOCs for exosome isolation through the integration of either “passive” or “active” components [131,132] for sorting. In “passive” chips, no external forces, excepting those needed for fluid pumping, are used to achieve separation, because of the integration of microfluidic components that are able to entrap exosomes via immunoaffinity/size-exclusion or to drive exosomes through specific streamlines. On the other hand, in “active” chips, beyond fluid pumping systems, electric or magnetic external sources are applied to sort nano-size objects. Both of the approaches are discussed in the following sections and the experimental details, advantages/disadvantages of each of them summarized in the Table 3 and Table 4. 

### 3.2. Passive Sorting

#### 3.2.1. Immunoaffinity-Based Methods

The most common “passive” approaches to exosome isolation are based on the immobilization of selected antibodies in the inner surface of microfluidic chips. The antibodies are able to interact with specific biomarkers present on exosome outer surfaces; hence, exosomes containing the target protein can be separated from mixtures of other EVs by flowing inside the chip. The geometry of the microfluidic network is designed to maximize the interaction between the target exosomes and the antibody-derivatized surface. In the pioneering work from Chen et al., the internal walls of herringbone structures, which are well known to enhance mixing capabilities, have been functionalized with CD63 antibodies (common exosomial markers) [133]. A serum aliquot was injected into the chip, and, from the trapped exosomes, about 30 ng of RNA were extracted. The quantity and quality of exosomal RNA recovered from the chip was enough to detect tumor-derived RNAs. The method resulted in more convenient than traditional technologies being faster and employing smaller amounts of reagents. Similarly, Fang et al. optimized on-chip immunocapture of breast cancer-derived exosomes from both cell culture medium and patient plasma [134]. 

**Figure 2:**
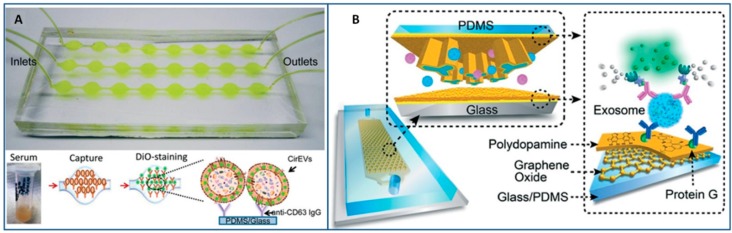
Immunoaffinity methods to trap exosomes. (**A**) Prototype of the Exochip and scheme of exosome immobilization through a CD63-antibody coating during serum flow. Reproduced with permission from reference [115]; (**B**) Nano-IMEX chip consisting of an array of Y-shaped microposts; focus on the functionalization with graphene oxide (GO) and polydopamine (PDA) to obtain an enzyme linked immune-sorbent assays (ELISA)-like assay with enzymatic fluorescence signal amplification. Reproduced with permission from reference [135].

As an evolution of Chen’s chip, Kanwar et al. developed the “Exochip” with a different design consisting of several circular capture chambers to further enhance mixing, and, consequently, the immunocapture efficiency based [115]. Exosome quantification was performed by fluorescence assays on a standard multiplate reader, being the chip designed to fit into them. A content of 15–18 μg of total protein and 10–15 ng of total nucleic acid was isolated from the treated samples and a higher level of fluorescence was detected in exosomes from pancreatic cancer when compared to healthy volunteers, in agreement with traditional assays (Figure 2A). 

With the aim of improving the sensitivity of the chips by reducing non-specific interactions, Zhang et al. integrated nanostructured coatings that are based on graphene oxide/polydopamine into the nano-Interfaced Microfluidic EXosome (nano-IMEX) platform [135]. A high mixing and exosome trapping efficiency with suppression of non-specific exosome adsorption was induced and an ultrasensitive exosome ELISA assay based on this interface was developed. As a proof of concept, the nano-IMEX platform was used to discriminate ovarian cancer patients from healthy controls (Figure 2B).

**Figure 3:**
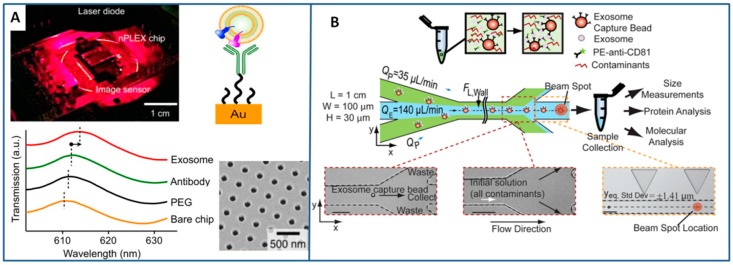
Immunoaffinity strategies to trap exosomes directly on the sensor flat surface or on beads. (**A**) Picture of the nPLEX imaging prototype; a scanning electron micrograph showing the array of nanoholes and the graph revealing changes in transmission spectra due to exosome detection. The sensing interface is functionalized by specific antibodies able to bind exosomes and stabilized by a polyethylene glycol (PEG) layer to minimize nonspecific interactions. Reproduced with permission from reference [136]; (**B**) Exosomes in the biofluid are pre-treated with capture beads. Then, the fluid is injected in the device with a co-flow of tris-buffered saline (TBS) at different volumetric flow rates (Q_E_ and Q_P_); beads migrate in the middle of the microchannel and contaminants are siphoned off under the effect of inertial lift forces (F_L_). Scale bar: 100 µm. Reproduced from reference [114], with the permission of AIP Publishing.

Alternatively, the immune-affinity isolation can be performed directly on the surface of transducers, like those that are based on surface plasmon resonance (SPR), thus eliminating the need to produce multichannel arrays to concentrate and isolate the largest amount of exosomes from a biological sample. With this aim, Im et al. produced an on-chip nano PLasmonic EXosome sensor (nPLEX) consisting of several nanohole arrays patterned on a metal film and functionalized with different affinity ligands [136] (Figure 3A). The chip was used to “map” the exosomes that are present in ascetic fluid from ovarian cancer and non-cancer patients. Although demonstrated only with exosome-rich samples (>109 exosomes per mL), this technology is expected to be promising for lower content exosomes biofluids if integrated to sample preparation modules. Similarly, a Surface Plasmon Resonance (SPR) platform was used to isolate an exosome population from a small cohort of breast cancer patient samples to identify that approximately 14–35% of them express the human epidermal growth factor receptor 2 [137].

Lastly, in other exosome separation methods that are based on immune-affinity, antibodies are immobilized on beads. If the beads have magnetic properties, “active” separation methods that are driven by external magnetic forces can be applied (see Section 3.3.2), otherwise inertial microfluidic systems are used. For instance, Dudani et al. bonded exosomes to polystyrene beads, and then optimized a microfluidic platform based on rapid inertial solution exchange to achieve 100% transfer efficiency of exosome capture beads from biofluids into a wash buffer [114]. After the immobilization, exosomes were eluted from the capture beads and characterized (Figure 3B). The device was tested with blood samples that were spiked with centrifuged melanoma and breast cancer culture supernatant.

A crucial point in immune-affinity assays is the interaction selectivity: non-specific interactions can be reduced by a proper surface functionalization with ordered self-assembled monolayers (SAMs). In this regard, β-mercaptoethanol and mercaptoundecanoic acid are often used on gold surfaces to reduce unfavorable biomolecular interactions [138,139], as well as silanes on paper substrates with improvements of detection limits, expandability, and adaptability of the method [140]. Although immunoaffinity-based methods for exosome isolation are the most used, they are limited to exosomes containing the target protein. Additionally, they are not selective with respect to the exosome sizes, which represent an important parameter for several biochemical processes.

#### 3.2.2. Size-Exclusion Methods

Size-based separation approaches incorporate nanoporous membranes or nanoarrays between or inside microfluidic networks. A first example of exosomes separation while using membrane technology was reported by Davies et al. [141]. A pressure-driven filtration process on membrane was implemented to separate small exosomes directly from mouse whole blood and remove debris and cells; little amount of filtrate was enough to assess the exosomal cargo by western blot and RT-PCR and the process was very fast when compared to other techniques. Successively, electrophoresis was also implemented, as discussed in Section 3.3.1, leading to improvements in the throughput and the purity of the collected exosomes.

Rho et al. used a microfluidic device consisting of a membrane filter and a capillary guide sandwiched between two ring magnets (that fix the filter inside the chip and allow for easy filter replacement after use) to isolate exosomes from unprocessed blood samples [142]. The capillary layer guides the filtered exosomes toward the collection channel. A single microvesicles population was recovered from 300 μL of sample; larger volumes of samples can be processed on the same chip after filter replacement.

In a recent work, Liang et al. implemented a double-filtration microfluidic system to isolate exosomes from urine samples [143]. The fluid in the chip was first filtered through a large pore size membrane to retain larger vesicles and impurities; the flow through a second membrane with smaller pore size allowed for protein removal, isolation, and enrichment of the exosomes with high throughput and good recovery yields when compared to ultracentrifugation. 

**Figure 4:**
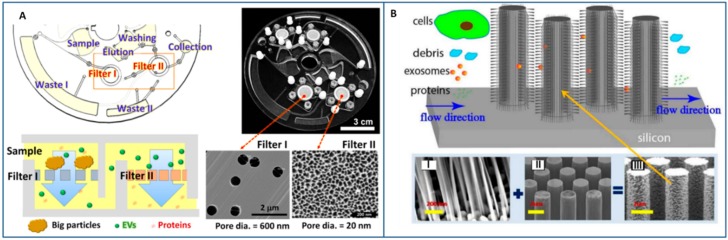
Size-exclusion methods for exosomes separation based on membranes and micropillars. (**A**) Photograph and schematic diagrams of the Exodisc showing its multiple functions of filtration, washing, reagents storage, and waste collection. Scheme and SEM images of the two filters (pore diameters of 600 nm and 20 nm, respectively) Reproduced with permission from reference [144], Copyright 2017 American Chemical Society; (**B**) Schematic of the trapping process of exosomes in a ciliated micropillar array and zoom of I) the porous silicon nanowire forest, II) micropillars, and III) ciliated micropillars. Reprinted with permission from reference [145] Copyright 2014 ROYAL SOCIETY OF CHEMISTRY.

A double filtration system was also implemented in a tabletop-sized centrifugal microfluidic system named “Lab-on-a-disc” for exosome isolation and analysis (Exodisc) [144]. In the Exodisc, Woo et al. used 20 two pore size membranes to isolate exosomes with low purity but with high throughput and high recovery yields, by removing >95% of protein contaminants. Furthermore, the mRNA concentrations of the isolated EVs were >100-fold higher than those that were isolated by ultracentrifugation (Figure 4A).

A step-by-step assembly from laser-cut plastic layers was used to produce the Exosome Total Isolation Chip (the so-called ExoTIC), a modular and easy-to-use platform that is based on a nanoporous filter membrane. The plastic housing of the membrane was secured with metal screws, nuts and plastic gaskets, to provide a leak-free connection and allow for fluid treatment at 5 mL/h. An amount of concentrated sample enough to allow subsequent analysis was collected from the filter membrane using a standard pipette within 2 h, while free nucleic acids, proteins, lipids, and other small fragments were flushed out. The chip efficiency was demonstrated by treating several cancer patient clinical samples with isolation yields that were ~4–1000-fold higher than ultracentrifugation [146]. 

Although not demonstrated with clinical samples but only while using phospholipidic “exosome-like” vesicles, it is interesting to mention the proposal of trapping exosomes into chips containing ciliated micropillars [145]. The ciliated structures consisted of microporous silicon nano-wires that are able to release exosomes by treatment in phosphate-buffered saline. The trapping process was relatively fast (≈10 min), with good retention yields (60%) for 83 nm vesicles (by processing tens of μL of sample. For larger sample volume, the retention rate was found to decrease probably because of saturation effects (Figure 4B). Finally, size-selective technologies allow for exosomes separation with high size uniformity and without the contamination of non-exosomial proteins or MVs, thus overcoming the main limitation of immunoaffinity. However, the application of size-exclusion methods appears still restricted because of their intrinsic saturation limits that increase the risk of clogging and decrease the recovery rates. Furthermore, the complicated photolithography fabrication processes often needed to produce nanoarrays as well as the non-continuous nature of size-exclusion separation methods also contribute to their scarce diffusion.

#### 3.2.3. Flow-Induced Methods

Exosomes sorting can also be achieved in microfluidic devices in continuous flow by taking advantage of the streamlines that particles are forced to follow because of physical constrains that are imposed by the chip design or by the mechanical properties of the mean. Three examples of these devices are based on Deterministic Lateral Displacement (DLD), Pinched Flow Fractionation (PFF), and viscoelastic microfluidics.

**Figure 5:**
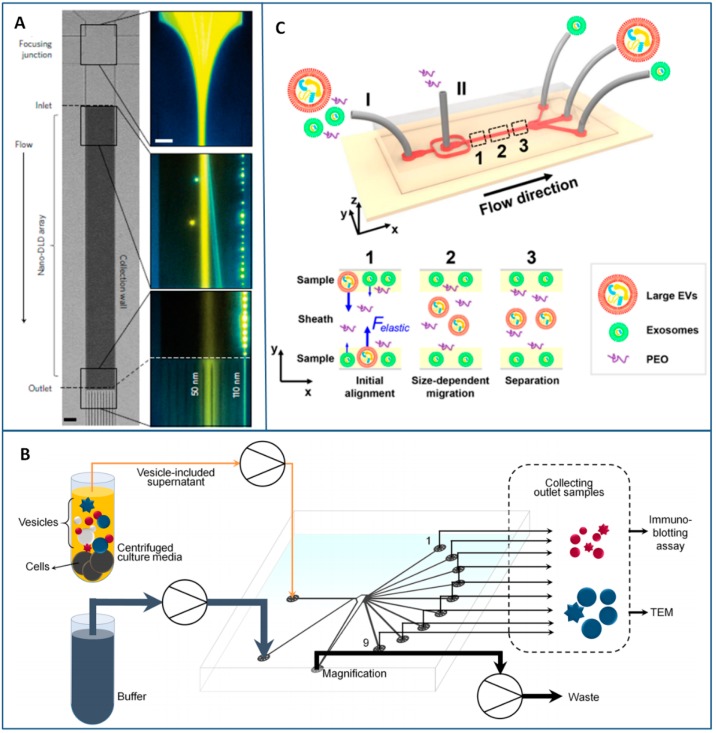
Flow-induced methods for exosomes separation based on Deterministic Lateral Displacement (DLD), Pinched Flow Fractionation (PFF), and viscoelastic microfluidics. (**A**) Hydrodynamically focused jet in a nano-DLD array first tested to separate polystyrene beads and the corresponding false-colour fluorescence images illustrating the separation process. Scale bar: 10 μm. Application of the chip to real samples (human urine) with a fluorescence-detection scheme [113]. Reprinted by permission from Springer Nature, Reference [113], Copyright 2016; (**B**) Biological vesicles from cell culture are collected after centrifugation and injected from one inlet of the chip, while the buffer is introduced from the other inlet channel. After separation, exosomes and apoptotic bodies are collected from the outlets and analyzed by immunoblotting assay and TEM. Reprinted from reference [150]; (**C**) Scheme of the microfluidic chip in which sample and sheath fluids containing low concentration of poly(oxyethylene) (PEO) are introduced from inlet I and inlet II, respectively. Large vesicles are collected at the middle outlet and exosomes at the side outlets because of the elastic forces (F_elastic_) acting during the flow, as shown in the scheme below. Reprinted with permission from reference [152], Copyright 2017 American Chemical Society.

In the DLD method, particles continuously flow through a gradient of pillar arrays whose geometry determines a critical cutoff diameter DC. Particles with a diameter larger than DC are displaced laterally following a bumping mode throughout the array, while smaller particles travel following the streamlines of the fluid, thus enabling particle sorting [147]. Inspired by DLD technology, Santana et al. produced a chip to sort microvesicles as a function of diameter from heterogeneous populations of cancer-cell-derived extracellular shed vesicles [112]. They found a “statistically-significant” variance between outputs, which indicated that EVs were concentrated in the target output. In another work, Wunsch et al. succeeded in using DLD platform containing pillar arrays with gap sizes from 25 to 235 nm to separate exosomes from 20 to 110 nm at low Peclet (Pe) numbers, although with low recovery rates [113] (Figure 5A). A complex photolithographic process was needed to produce the pillar array as well as a surface modification of the device to avoid clogging.

In PFF, solutions of particles and buffer are respectively injected from two separate inlets in the chip and travel at different flow rates until reaching a pinched segment where the particles are pushed towards a side wall and they are constrained to move on streamlines defined by the positions of their centers of mass (which varies according to their size) [148]. Then, the solution moves towards a broadened segment of the device where the various streamlines (and particles within) are separated and collected from different outlets. The separation effect in the broadened segment can be further amplified by introducing a side channel that induces an asymmetric distribution of the flow [149]. The use of a PFF with nine outlets and a tunable magnification side channel for EVs size based-separation was recently reported by Shin et al. [150] (Figure 5B). A fine control of particle allocation inside the broadened segment and, hence, in the outlets was achieved by tuning the flow rates of the two inlets and by modulating the withdrawing flow of the magnification channel. The efficiency of the device was demonstrated by separating nanometer-sized exosomes and apoptotic bodies from cell culture media. Unfortunately, tests on complex biological samples, like blood, were not performed, but the authors believe that the application of such chip to real samples is possible and very promising.

Another approach to flow-driven separation of exosomes is based on viscoelastic microfluidics that takes advantages from the different lift forces acting on particles of various sizes flowing in a viscoelastic medium [151]. A viscoelasticity-based chip was reported to directly separate exosomes from cell culture media or serum in a continuous, size-dependent, and label-free manner while using a small amount of biocompatible polymer as additive in the media [152]. Liu et al. demonstrated that exosomes of size below 200 nm in untreated fetal bovine serum are shifted by elastic lift forces along the sidewalls of the microchannel in optimized poly-(oxyethylene) concentration and flow condition; on the other side, larger vesicles tend to move towards the channel centerline (Figure 5C). Lastly, exosomes have been isolated from two-side outlets of the chip and larger vesicles from the outlet in the middle. For its simplicity and throughput (200 μL/h) viscoelastic microfluidics appears a promising approach, although further validations with clinical samples are necessary.

As a final consideration, flow-induced microfluidic approaches are dynamic methods to separate exosomes with high size uniformity and high purity while using very low volumes of samples. Some of them, like PFF and viscoelasticity-based chips, are also easy to fabricate and with low risk of clogging. DLD, instead, consists of nano-scale structures, and, therefore, has limitations that are similar to size-exclusion methods. A general disadvantage of all flow-induced methods is that they are based on microfluidics rules; hence, physical knowledge is needed for the design and operation of these devices. General features of these methods are summarized in Table 3.

### 3.3. Active Sorting

#### 3.3.1. Electroactive Separation

The sorting of extracellular vesicles (or circulating tumor cells) from a heterogeneous sample may be also achieved by active microfluidics approaches, which allow for exploiting different physical characteristics instead than simple size or immune-affinity. This further capability can facilitate the preservation of vesicle integrity or the isolation of subpopulations not recognized by antibody affinity or other biochemical methods. 

**Figure 6:**
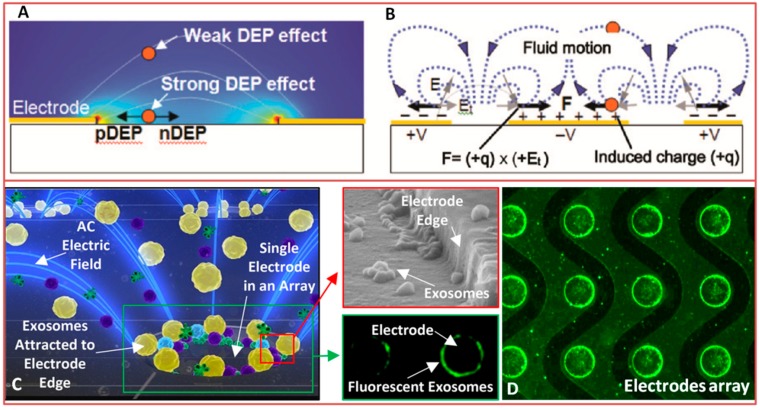
Schemes of two of the main electrokinetic behaviours (**A**) Dielectrophoresis; (**B**) Electro-osmosis. Figure reproduced with permission from reference [155]; (**C**) AC separation of exosomes through an array of circular electrodes confirmed by SEM and fluorescent acquisitions (**D**) Reproduced with permission from [165].

Electroactive strategies permit electro-osmotic flow without the need of pumps or other moving parts and electrophoretic sorting on the basis of different charge-to-mass ratios [153]. Numerical simulations are often employed to guide device design in a wide range of operational conditions [154] (Figure 6A,B). As a basic principle, electro-osmosis allows for driving a bulk flow by the interaction between an electric field and the electrical double layer (EDL) at the electrode surface. If the electric field contains a tangential component (Et), the ions in the EDL traverse laterally dragging the fluid along (Figure 6B). In the case of planar electrodes, this induces a continuous and rotational fluid pattern that can be described as a set of counter rotating vortexes and may cause particles to orbit out of plane or accumulate near the electrodes94. Recently, Dubov and co-workers exploited electro-osmotic flow anisotropies in a channel with grooved walls to achieve a lateral segregation of a uniformly mixed colloidal suspension (particles used had diameter of 200 nm and 2 μm in suspensions showing different conductivity). In this case, the recovery of particles with larger diameter resulted easier to be achieved [155].

Dielectrophoresis (DEP) occurs instead in the presence of a non-uniform electric field and enables separation that is based on dielectric constant differences that determine how quickly particles/vesicles move. The net drag force is either in the direction of higher (positive DEP) or lower (negative DEP) electric field strength depending on the relative polarizability between the particle and the surrounding media. Particle sizes and electric field frequency/strength influences the process. Progresses in electrode microfabrication allow today for an easy generation of the large field gradients necessary for DEP. Advantages of this approach are the DEP ability to both attract and repel particles, the few requirements in terms of instrumentation [156], the fact that no prior tagging is required for manipulation, and noninvasiveness (particularly important for cell sorting tools). Markx et al. [157] employed negative DEP to separate cells according to their dielectric properties and obtain different dispersion patterns. A commonly used approach [158] is to employ positive DEP to hold on the cells of interest, while the rest is flushed away and then release the cells for further handling. However, this results in releasing discrete batch of sorted cells, thus efforts have been dedicated for developing continuous cell sorting techniques. In this respect, Narayanan and coworkers combined DEP with split flow thin fractionation (SPLITT) to separate nanoparticles with high precision and in a continuous manner based on their size, electrophoretic mobility and propensity to hold a precise streamline (a lower voltage was reported for moving 220 nm amino-coated particles than for the 108 nm amino-coated particles) [159]. Previously, Doh and Seger [160,161] exploited DEP to move cells from one laminar flow to another one. Recently, Mohammadi et al. achieved plasma separation from a blood droplet at low voltage, taking advantage of the hydrodynamic effect to improve red blood cells (RBC) trapping [162]. 

Other manipulation techniques include three-dimensional (3D) field-cages, where the electrode configuration generates a local three-dimensional field in which cells can be trapped and confined, as well as travelling wave DEP [163] where a travelling electric field moves the cells in a conveyer belt-like fashion [164] . By this technique, larger DEP force is transferred to samples due to gradient electric field between 3D electrodes. The higher gradient forces separation and trapping.

At reduced scales, in 2017, Ibsen et al. achieved on-chip separation of glioblastoma exosomes spiked in human plasma, exploiting differences in their dielectric properties. Notably, the application of an AC field allowed for a quick isolation and collection in less than 30 min. The entrapment of exosomes and MVs on the microelectrodes was confirmed by SEM analysis and on-chip (in situ) immunofluorescence with specific CD63 and TSG101 tagging proteins (Figure 6C,D) [165]. More recently, Shi and collaborators realized a nanopipette-tool that is able to capture nanoparticles from a close proximity region in front of the pipette’s tip, while using a DEP approach with a low DC field [166].

#### 3.3.2. Immunomagnetic Isolation

Magnetic separation is largely employed for differentially sorting specific blood cellular elements (RBC, leucocytes, etc.), as well as MVs and exosomes. Magnetophoresis is a simple and non-invasive method and has been described as one of the most efficient to enrich for exososmes if compared with standard techniques, like differential centrifugation and density gradient isolation. After conjugation of superparamagnetic beads with capture probes and molecular recognition of the target analytes, the magnetized bio-conjugated beads can be easily extracted from the non-magnetic matrix by means of an external field [167]. Notably, as the magnetic force is not directly applied on the cells, this minimizes the possibility of induced damages and the reduction of cell viability, which is typical with dielectrophoresis. As further advantages, magnetophoresis generally avoids solution heating and it is not influenced by solution pH and ionic concentration. 

**Figure 7:**
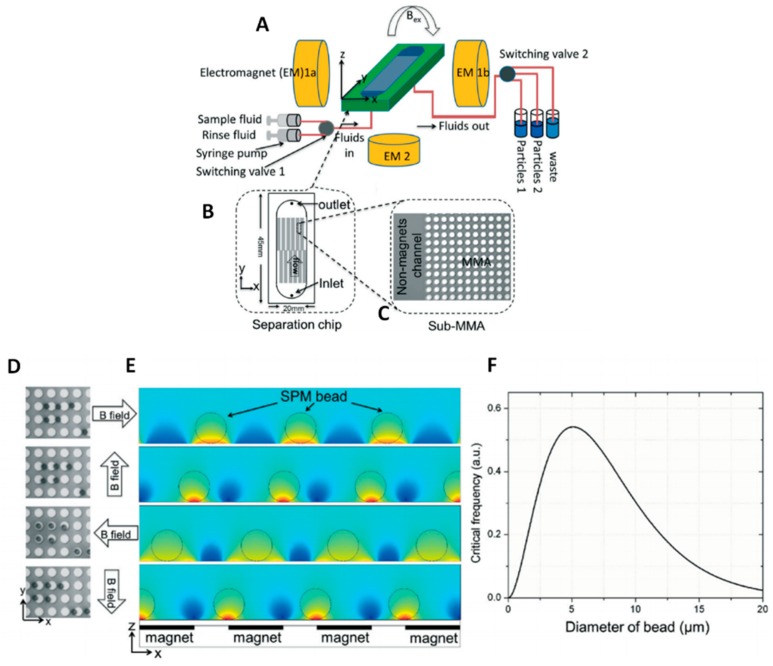
Scheme of the flow enhanced non-linear magnetophoresis system. (**A**) Non-Linear Magnetic separator components including a separation chip (**B**) with a micromagnet array with non-magnetic channels; (**C**) a set of programmable electromagnets (EM 1 and 2) for the generation of the rotating magnetic field, syringe pumps, switching valve, and fluid collection system; (**D**) Non-linear magnetophoretic transport of 5 μm superparamagnetic beads on a micromagnet array by optical micrographs at four different angles of external magnetic field; (**E**) Theoretical model for magnetic potential energy distribution on the micromagnet array in the presence of magnetic beads. The warmest colours identify lowest potential energy areas where the beads move towards; (**F**) Graph of the theoretical critical frequency as a function of the beads diameter for the cobalt micromagnets. Figure rearranged from reference [168] with permission.

Beyond magnetic susceptibility, the particle radius is another key parameter in determining magnetophoresis separation and it changes if biomolecules, microvesicles, and/or exosomes bind to the surface of beads. Following this general principle, the group of Li [168] moved (along × direction) superparamagnetic 5.0 μm beads by means of an external rotating field until the edges of a micromagnets array made of 100 nm-thick cobalt discs. Separation was then achieved along the transversal (y) direction due to hydrodynamic forces, with proof of concept being demonstrated by the isolation of exosomes from in vitro cultures of pancreatic cancer cell lines using coated magnetic beads (Figure 7).

In recent literature, several magnetophoretic approaches have been used both for the purification or for the enrichment of samples by using functionalized magnetic beads. As an example, Tauro and co-workers employed microbeads functionalized with monoclonal antibodies against the exosomal membrane surface protein EpCAM to capture exosomes derived from human colon cancer cell line, demonstrating a superior (at least twofold) capability of this method to enrich exosome markers and exosome-associated proteins in collected samples, when compared to other two biochemical methods, such as gel electrophoresis and Western Blot analysis [169]. Finite element simulations can again contribute in optimizing the procedure evaluating the trajectory of magnetic bio-conjugates in magnetic cell separation systems and the experimental performance with cells of varying magnetic susceptibility also providing guidance for scaling up [170]. 

A very efficient capture tool that is capable of high yields in exosome separation (>93%) was then developed by Shao and co-workers [171], who realized an integrated platform for the complete analysis of exosomes derived from glioblastoma cell lines. Cancer exosomes were captured in serum onto magnetic microbeads containing affinity ligands, like anti-CD63 and anti-EGFR. In subsequent modules of the platform, the immuno-enriched exosomal population was then lysed and analysed for their RNA content. More recently, a new platform, called ExoSearch, has been reported for the diagnosis of ovarian cancer in blood taking advantage of multiplexed measurements of three exosomal tumor markers (CA-125, EpCAM, CD24) while using a training set of ovarian cancer patient plasma, and showing diagnostic ability comparable with standard Bradford assay. Specifically, while using an immunomagnetic approach, a plasma sample and functionalized beads were introduced and uniformly mixed through a long serpentine channel to facilitate exosomes binding. Magnetic beads with captured exosomes were then retained as a tight aggregate in the downstream microchamber by magnetic force, allowing for the following steps of quantitative isolation and detection of the exosomes [172]. In this last case, authors tested their platform directly on plasma samples, obtaining results that were comparable with standard Bradford assay. 

The possibility to carry out separation of red blood cells without a magnetic label but on the basis of intrinsic magnetic properties due to iron in the haemoglobin was also reported (as well as for some strains of Bacillus spores due to manganese content) [173,174]. In this case, RBCs in suspension migrate along the magnetic field gradient toward the magnetic deposition zone. To this aim authors optimized a set up characterized by a fringing magnetic field at the interpolar gap, which was combined with a thin flow channel being pressed against this gap to obtain very high magnetic drag forces. Avoiding the need of labelling with magnetic beads can represent a significant advance in term of process simplicity, but in this case, forces acting directly on the cells/vesicles are introduced with potential risks on their viability/stability. Moreover, the presence of magnetic actuators allows for multiple reagent and washing streams, resulting in a large reduction in processing times. On the other hand, when dealing with magnetic beads as capture probes, the strong particle-particle interactions often create stable aggregates which hinders their further utilization. In addition, the entrapment of nonspecific impurities of sample in the capturing regions might affect the performances of the device, in terms of recovery efficiency and purity, making this approach suitable only for certain analytical applications [175].

#### 3.3.3. Acoustofluidics

Acoustophoresis is a biocompatible, non-contact, and label-free approach for manipulating particles and cell populations, which does not require modification of the media containing the analytes to be separated, a useful characteristic for maintaining cell/vesicles in their native states for following steps of culture and/or analysis. Acoustophoresis is based on the application of acoustic pressure fields by bulk acoustic waves (BAWs) or surface acoustic waves (SAWs). To generate the acoustic waves, piezo-actuators are typically employed, consisting of electrodes on a piezoelectric substrate, like lithium niobate or quartz. Due to piezoelectric coupling, the application of an alternating voltage among top and back electrodes generates mechanical vibrations in bulk ceramics in the form of standing bulk acoustic waves between a piezoelectric transducer and an acoustic reflecting interface, which are then coupled to the fluid within the microchannel. SAW instead propagates along the surface of the piezoelectric substrate with an amplitude decaying exponentially with the depth in the substrate and a wave energy confined to the substrate surface; for their excitation, interdigited electrodes are fabricated. 

Under the effect of acoustic pressure fields, particles experience differential forces according to their mechanical properties (size, density, compressibility) [176,177]. As a result, in an acoustic standing wave field, the acoustic radiation force induces small particles having a mass density that is greater than the medium to accumulate at the pressure nodes of the standing waves typically located in the microchannel centre, thus permitting particles concentration/separation. The efficiency of concentration and separation is greatly dependent on the spatial location of the pressure nodes, and thus the relative positions between the microchannel and the transducer must be precisely aligned. Other parameters to be considered for SAW are the geometry of interdigited electrodes [178] or the angular tilting of interdigited electrodes with respect to the microfluidic channels alignment [179]. In general, these technologies are suitable for miniaturization and to be easily incorporated into microfluidic networks. 

Currently, the number of applications of acoustic waves for bioanalytical and clinical purposes is significantly increasing, including tools for cell handling and sorting, as well as for spatial control and the manipulation of particulate matter in fluids and droplet generation and movement [180,181]. For example, the differentially sorting of blood elements based on their size or circulating tumor cells from blood samples were reported [182,183], as well as the implementation of the so-called “acoustic tweezer method” enabling confinement in specific positions (nodes and antinodes) into a microfluidic device [181]. 

**Figure 8:**
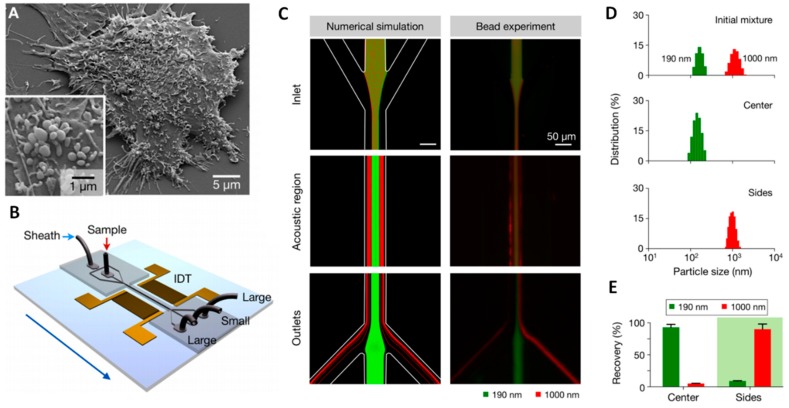
(**A**) MVs released by a human brain tumor cell; (**B**) Scheme of the acoustic nanofilter device in which a pair of interdigitated transducer (IDT) electrodes are used to generate a standing surface acoustic wave across the flow direction. In this configuration larger MVs are conveyed to the two side outlets, while the smallest were focused to the central one; (**C**) Numerical simulations are in agreement with the experiment for filter validation with polystyrene fluorescent particles of different size (d = 190 nm, green; d = 1000 nm, red); (**D**) Dynamic light scattering measurements confirm the size-selective enrichment of particles and the recovery rate (**E**) was estimated as >90% for both types of particles. Adapted with permission from reference [116]. Copyright 2015 American Chemical Society.

Despite the relatively high number of applications of acoustic waves in separating elements from clinical samples, the use of SAW-based devices that are focused to the direct sorting of microvesicles or exosomes is a less explored field. Only few examples are reported in recent literature. One of these includes the design of an analytical model predicting the trajectory of 200 nm and 1 μm diameter particles in a viscous medium, and the transit time of simulated exosomes moving from the core stream to the sheath flow when the SAW is applied. Authors also realized the microfluidic devices and tested them with polystyrene beads and as an acoustic nano-filter to enrich exosomes from different types of EVs [116] (Figure 8). 

**Figure 9:**
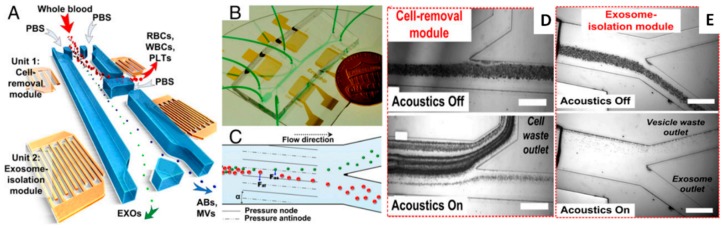
(**A**) Scheme and mechanisms of the acoustofluidic device for isolating exosomes. Red blood cells (RBCs), white blood cells (WBCs), and platelets (PLTs) are removed in the first separation step and then subgroups of EVs (microvesicles, apoptotic bodies and exosomes) are sorted by the exosome-isolation module; (**B**) Optical image of the setup developed by Wu and co-workers; (**C**) Schematic of size-based separation due to the periodic distribution of pressure nodes and antinodes in a surface acoustic waves (SAW) field. Larger particles are pushed toward node planes; (**D**,**E**) Optical images of the separation steps corresponding to the elimination of blood elements and exosomes collection (Scale bar: 500 μm). Figure rearranged with permission from reference [184].

More recently, Huang and co-workers realized an acoustofluidic platform that is able to isolate exosomes directly from undiluted whole-blood samples thanks to the integration of two sequential surface acoustic wave (SAW) microfluidic modules. The first is the cell-removal module that extracts microscale blood components that are larger than 1 μm in diameter, including red blood cells, white blood cells, and platelets, in order to obtain a sample enriched in EVs. Through the exosome isolation module, by using a higher frequency (~40 MHz) than those that were used in the first one, authors achieved the removal of EV and apoptotic bodies, obtaining a sample with purified exosomes. Each module relies on a tilted-angle standing SAW field [184] (Figure 9). 

In Table 4 a summary of the available technologies for active separation methods, encompassing pros and cons of each of the active methods under consideration is reported. Electroactive, magnetic and acoustic technologies allow for the high performance sorting of micro and nanoparticles and some examples in literature are reported, dealing specifically with exosomes. Electroactive methods represent probably the easiest way to obtain miniaturized devices, due to the availability of micromanufacturing technologies. Close to this advantages, however, the needing for heavy benchtop instrumentation is required to generate electric field for actuation. As a drawback, an undesired heating of the solution was sometimes reported [155]. When considering this aspect, magnetic actuation may fulfil the requirements for both miniaturization and portability, as the application of a magnetic field does not require large and expensive instruments. Moreover, magnetic labelling of the biological entities does not affect the viability of a sample and it is easy to be achieved, even the labelling of capture probes usually implicate additional costs. SAW-based devices have strong potential for both portability and high-performances, but piezoelectric substrates costs and instruments that are required to generate surface acoustic waves limit their spreading in common use and research.

### 3.4. Discussion

After reviewing the innovative methods being reported for EVs sorting, it is worth drawing some technical considerations. A number of (passive/active) microfluidic technologies are today available and mature enough for providing next generation clinical tools that are able to perform the enrichment of biological samples for a particular species. Not all of the considered technologies have been applied for sorting exosomes, and transitions from cells and MVs to the nanoscale is not an automatic process, but extension to exosome field may be expected by further tailoring of process parameters to match the needs of the specific application. When compared to standard methods, miniaturiaztion in lab-on-chip platforms directly results in lower sample consumption per analysis (resulting in an improved patients’ compliance) and cost reduction with respect to benchtop instruments. Additionally, LOCs enable increased automation and customization by exploiting dedicated MEMS technologies, allowing also for advances in terms of performance (recovery yield, purity, and size uniformity) and accuracy toward precision medicine. As a further advantage, even if EV analysis is not expected to be run at the point of care or by common people, highly trained personel is not required with miniaturized tools because of the automatization of clinical tests, favouring the spreading of personalized therapy approaches also within peripheral laboratories.

## 4. On-Chip Counting Modules

The number and content of MVs and exosomes were reported to change in the case of pathological conditions and demonstrated to be valuable biomarkers for diagnosis and prognosis of different disorders. Thus, it is worth reviewing also the available approaches to address them after the sorting process. For the enumeration and analysis of sub-populations of cells, circulating vesicles, and/or micron-sized particles into a complex and heterogeneous matrix, like blood, flow cytometry [187] still remains the gold standard, with many examples of innovative cytometric methods being optimized through years in order to improve experimental performances [188,189]. For transduction, optical and electrical detection systems are typically employed, but sometimes they were also combined together. 

Basic principle of particles counting methods relies on the counting of pulsed signals corresponding to transit and specific detection of single objects flowing in a microchannel. Single or multiple fluorescent tags for membrane markers of the researched species are the key components of optical approaches. Antibodies against surface membrane markers, like Anti-EpCAM, Anti-EGFR, Anti-CD63, or Anti-CD9 [136,169] are some of the most employed labels, and, after specific recognition with their counterparts, allow for the detection and counting upon laser excitation and detection. 

Miniaturized flow cytometers have been realized through years in order to improve portability and ease of use of a common tool for cells analysis and to shift from full-sized laboratory benchtop instruments which are expensive and usually dedicated to limited experiments to more compact platforms that are able to perform high throughput analysis in an automatic manner [190]. Recently, for example, the group of Russom developed an all-silica fibre microflow cytometer that detects fluorescence and scattering from particles and cells. It integrates circular capillaries for cell transport and optical fibres for light delivery. Single particle focusing is performed exploiting elasto-inertial microfluidics considering flow rates up to 800 µL/min and enabling detection of 2500 fluorescent particles per second [191].

An affinity-based scheme (using antibodies against surface markers) was employed by Daaboul and co-workers, who recently employed interferometric reflectance imaging sensors for enabling multiplexed phenotyping and digital counting of subpopulations of individual exosomes captured on a microarray-based solid phase chip [192]. Notably, in this work, size discrimination and counting of single particles was brought to a new limit, down to 50 nm, a too small dimension to be accurately detected by conventional methods, such as optical microscopy and flow cytometry. The vesicle binding to the capture antibodies was evaluated by subtracting the signals measured before and after sample incubation, with spatial location in the array encoding information on the exosomes’ phenotype. An image processing software was used to quantify relative nanovesicles size [192].

Beyond optical signals, electrical approaches were also proposed and more recently implemented. In this respect, the use of the so-called “split ring resonators (SRR)” looks very interesting and promising for the high sensitive and real-time detection of circulating particles/cells and have the potential for high degrees of miniaturization [193] and integration into microfluidic platforms [194]. The idea is to fabricate a circuit having a split ring shape that is characterized by a microwave resonance spectrum and an electric field localized in the gap. The equivalent electrical circuit includes the capacitance of the split region that is very sensitive to changes in the dielectric properties of the material surrounding and within the gap, such as those that are related to the transit of a particle/cell [195]. If a high Q factor is achieved, then high sensitivity can be enabled. In this respect, Zarifi and collaborators developed an approach based on a high resolution planar resonator sensor able to perform non-contact particle size classification in both liquid (water as a lossy environment) and gas (air) phases using petroleum coke beads as a test material [196]. Cytometry applications of SRR are still a quite unexplored frontier.

As far as the hybrid approaches are concerned, Morgan et al. recently developed a microchip which integrated together single particle fluorescence spectroscopy and multifrequency electrical impedance sensing using an AC signal of a few hundred millivolts. Specifically, in their setup, metal electrodes similar in size to a cell (typically around 10 μm) were fabricated and precisely aligned at the bottom of microchannels and two laser beams were focused at the mid-height of the channel. When a labeled particle (or a cell) comes across the detection window, impedance changes and contemporary the fluorescent emission is detected by the optical system [197]. Another similar approach combines optical detection with MOSFET (metal oxide semiconductor field effect transistor) technology in a setup where the MOSFET gate is coupled to a sensing aperture in the fluid circuit. If a microparticle, which is less conductive than the suspending medium, passes through the aperture, it determines a gate voltage change that is further amplified by the MOSFET as a drain current drop. In this way, authors were able to distinguish particles from 2 μm to 15 μm, on the basis of the resistive pulses and thus to count flowing objects. The counting of the resistive pulse events gives information about the total number of cells passing through the sensing aperture, while the fluorescence detection system determines only the number of fluorescent-labeled CD4+ T cells, providing information about the prevalence of a specific subpopulation of lymphocytes [198]. 

## 5. Exploring the Content of Vesicles

As discussed before, numerous studies have demonstrated the huge potential of MVs and exosomes as diagnostic, prognostic, and even therapeutic entities to transport drugs. Moreover, they have an important role in the spreading of diseases throughout the body districts [3]. During their biogenesis, microvesicles and exosomes are packaged with proteins, RNA and lipids partially reflecting the content of their cells of origin, and become highly stable reservoirs of disease biomarkers. Being able to travel via body fluids and be easily internalized, they can transfer their content to recipient cells and for this reason exosomes have been defined as the “Trojan horse” of the intercellular communication [199,200]. In some cases, proteomic and genetic investigation also showed distinctive profiles for the extracellular vesicles when compared with whole cell lisates, which could be instrumental for disease progression, making it reasonable to assume that vesicle production allows for the primary site of the diseases to exert different effects, according to the possible acceptor targets. For instance, in tumour diseases, vesicles have been investigated for their potential to enhance the malignant properties of adjacent neoplastic cells or activate non-tumoral cells [201]. 

As a result, apart from their identification and counting, a great diagnostic significance relies in MVs and exosomes content and it is of crucial importance to determine their cargo of proteins, functional messenger RNAs (mRNAs), and microRNAs (miRNAs) [202] and double-stranded DNA (dsDNA) (which may include oncogenes). Knowing and cataloguing the exosomal proteins, RNAs and lipids may be a very useful tool in order to gain deeper insight about their role in bearing information among cells, as well as disease biogenesis and progression. To meet this need, an online database that catalogues exosome specific data concerning proteins, RNAs, and lipids, named ExoCarta [203] (http://www.exocarta.org/), has been also created as a highly accessible website to compare sequences and upload new ones. 

### 5.1. Lysis Module

Once isolated and counted, MVs and/or exosomes should be characterized in order to achieve a complete analysis and maximize the acquired information. For this purpose, breaking their external membrane is required to allow for the consequent release and gain accessibility to their content. However, being a disruptive event, membrane lysis methods need to be appropriately tailored in order to keep intact the components (such DNA, RNA, and proteins) for further analysis. 

**Figure 10:**
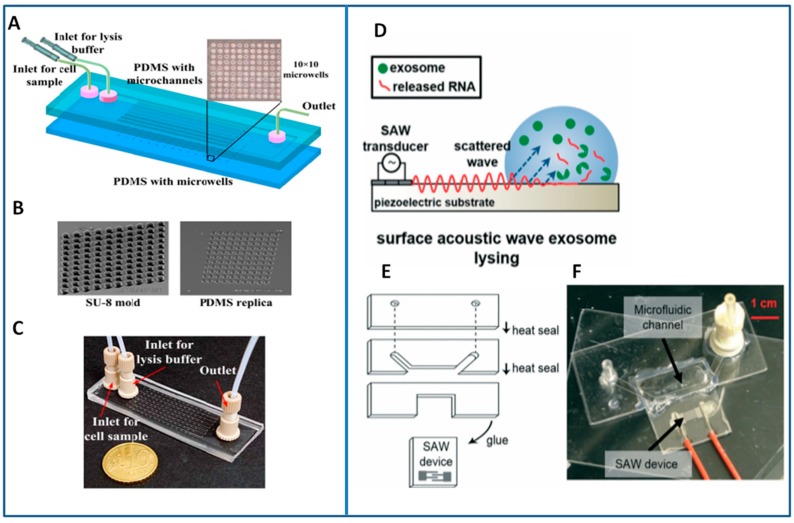
(**A**) Diagram of the microfluidic chip proposed by Jen and colleagues able to sort single cells thanks to polydimethylsiloxane (PDMS) microwells (**B**) obtained by soft lithography from an SU8 hard master; (**C**) Picture of the chemical lysis device reproduced with permission from reference [208]; (**D**) Operation mode of the SAW-based lysis module developed in the work of Taller et al., made by a multilayer structure (**E**) of polycarbonate on a Lithium niobate substrate with SAW interdigited electrodes; (**F**) Picture of the exosomes lysis module D, E, and F portion reproduced with permission from reference [211].

As starting point, the approaches that were developed for cell lysis are available. They are based on either chemical [204] or physical (electrical [205,206], magnetic [207]) methods, and may be potentially applied also to the membrane of MVs or exosomes. Commonly used chemical lysis buffers include sodium dodecyl sulfate (SDS), proteinase K or Triton X-100 even though a differential efficacy has been reported on various target subpopulations: for example, MVs and apoptotic bodies were found to be more sensitive to detergent lysis than exosomes [2]. Although the chemical method requires some longer time to be applied, it is very simple and cheap, preserves the integrity of subcellular components even though marginally damages DNA. Recently Jen and co-workers realized a microfluidic chip with microwells of few tens of μm in diameter, which is able to perform chemical lysis for human carcinoma cells at the single-cell level [208]. They passively confined at a very low concentration cells inside microwells through microchannels that gradually settle cells toward the bottom surface following streamlines, leading into the microwells and, after cells were positioned into the microchambers, a lysis buffer was injected into the chip and the effectiveness of the lysis process was monitored by fluorescent calcein detection (Figure 10A–C). 

On the other hand, a common physical method for achieving on-chip lysis is the application of an external electric field that induces changes in plasma membrane, causing the production of holes on the cell membranes with consequent release of intracellular materials. The applied field has to be chosen carefully in order to exploit differences in transcellular-membrane potential (around 60 mV) and transorganelle-membrane potential (around 160 mV for mitochondria). This can be easily achieved on a chip thanks to microfabrication techniques [206]. 

Exosomes can present specific needs for lysis being an order of magnitude smaller than most cells [209]. Recently, Taller and collaborators achieved a rapid on-chip lysis of exosomes (in 30 min) and the detection of the released miRNA by applying surface acoustic waves. Specifically, transducer electrodes were realized on a piezoelectric Lithium Niobate substrate and channels for fluid flow were fabricated while using three layers of polycarbonate and aligned with the SAW device component. Sample (100 μL) containing exosomes from a pancreatic cancer cell line has been then pumped through the microchannel allowing for a residence time in the SAW exposure area of around 30 s (at 1 W of power). Beyond inducing an effective turbulent mixing, the electromechanical coupling that is achieved through the SAWs produces an electric field as high as ≈106 V/m at the substrate surface. The lysis of smallest entities like exosomes is made possible by the effect of the acoustic radiation and the dielectrophoretic force acting on small particles [210]. The efficiency was estimated as a lysis rate of 38 ± 10% while considering also that 10% of the exosomes are lost, being probably captured by the sticky microchannel walls [211] (Figure 10D,E). 

### 5.2. On-Chip Detection of Nucleic Acids

When dealing with nucleic acids, which are also conveyed from EVs and may work as carrier for mutations with a role in carcinogenesis, a convenient strategy is typically to exploit amplification methods for generating multiple copies of a target nucleic acid, either through standard PCR cycles or alternative approaches, such as isothermal amplification [207,212] and subsequently analyse the sequence looking for specific genes or point mutations. However, off-chip nucleic acid-based detection relies on complex analytical procedures and requires a relatively long time with multiple steps, expensive instruments and reagents, and trained personnel. In this respect, on-chip amplification and detection of nucleic acid is promising for improving performances, beyond enabling point-of care analysis, by integrating all of the required functional steps in a single miniaturized cartridge [213]. 

Various tools for nucleic acid analysis have been developed considering different approaches. One of the most exploited transduction methods is based on the integration of optical detectors, which are able to provide sensitive and accurate analysis of the amplification process. The most popular strategies take advantage of the integration into transparent platforms of light emitting diodes (LED) for illumination and low cost CMOS web-camera for optical detection [214]. Recently, also innovative methods to improve the performance of PCR have been miniaturized in biochip platforms. Myers and co-workers for examples developed a microfluidic Biomolecular Amplification Reader (µBAR) which is able to perform isothermal nucleic acid amplification assays and integrates a real-time fluorescence readout while using LEDs and phototransistors, significantly reducing the cost of conventional benchtop thermocyclers. The device includes also a temperature control module through an integrated resistive heater and monitors real-time fluorescence signals from 60 individual reaction chambers. Assays are carried out on PDMS disposable microfluidic cartridges [215]. Also, droplet microfluidics are on the rise for their strong potential of improving digital PCR capabilities in miniaturized devices. Some years ago, Hatch and collaborators developed a multi-channel device for the high-throughput automation of analysis in continuous flow by droplet microfluidics. In this work, authors demonstrated real-time qPCR in 0.1–10 μL droplets in a range of seven orders of magnitude concentration of expected starting nucleotides (from 1 × 101 to 1 × 107) [216]. 

**Figure 11:**
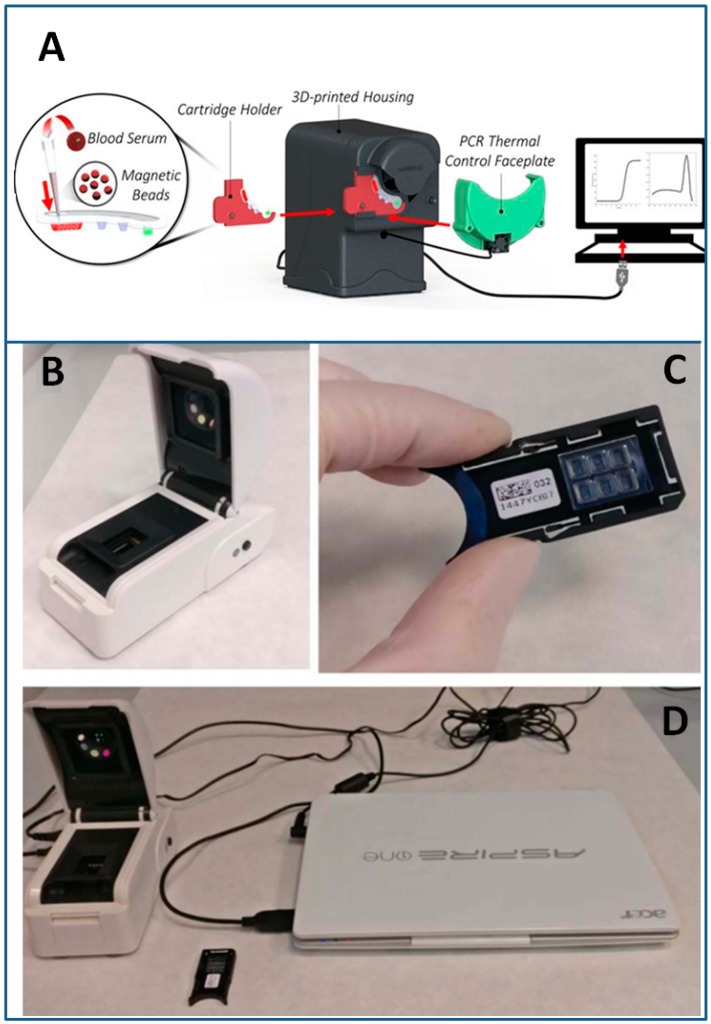
(**A**) A sample-to-answer qRT-PCR system for the detection of RNA from Hepatitis C virus (HCV) developed by Shin and co-workers using a droplet magnetofluidics tool in plastic cartridges [217]; (**B**–**D**) Q3 compact platform developed by Biava and co-workers for the real-time detection of viral nucleic acids extracted from plasma, urine, and oral swabs. Reproduced with permission from reference [220].

Recently, Shin and co-workers developed a sample-to-answer qRT-PCR system for RNA from HCV, utilizing a droplet magnetofluidics tool in plastic cartridges. Taking advantage of a precise and rapid thermal control of the system, droplet magnetofluidic sample processing enables the necessary purification of nucleic acid targets from clinical samples, in order to obtain quantitative and consistent assay results. To allow the automated PCR analysis, the cartridge consists of four wells preloaded with droplets of a binding buffer, two wash buffers and a PCR reaction mix. The presence of pH-responsive magnetic particles in the first well, combined with the acidic binding buffer, induces a positive charge on the polyhistidine coated magnetic particles. Negatively-charged nucleic acids in solution bind with the magnetic particles so that they can be subsequently transferred into the PCR well, through the application of a magnetic drag force. An optical detector integrated for PCR fluorescence acquisition revealed a high analytical sensitivity with HCV RNA positive samples ranging from 6,000,000 IU/mL down to approximately 45 IU in serum (equivalent to 4500 IU/mL) with good correlation with standard laboratory methods for PCR [217] (Figure 11A). Based on an optical detection method and a silicon disposable lab-on-chip cartridge, Biava et al. used a highly compact platform [218,219], for the real-time detection of viral nucleic acids extracted from plasma, urine, and oral swabs [220] (Figure 11B–D), resulting in a robust and easy-to-use tool. 

Non-optical transducers for the highly sensitive detection of nucleic acids are less widespread and include the electrochemical detection of RNA [221] or miRNA, as developed, for example, by Hou and collaborators. Specifically, they realized a tool to detect a hybridization chain reaction (HCR) amplification [222] by designing two DNA hairpins partially complementary to the miRNA sequence under investigation. Combining the hairpins with quadruplex DNA molecules that are able to form long chains, they obtained the amplification of the signal above ITO electrodes employed for the electrochemical detection [223]. Other approaches that are reported in literature include the use of ion-exchange nanomembranes realized to detect RNA [211], Field-Effect Transistors for the rapid, and the ultrasensitive detection of miRNA [224]. The realization of DNA arrays based on electrical nanojunctions shorted when nucleic acid targets conjugated to conductive nanoparticles bind to capture probes that are immobilized on nanoelectrodes [225]. In this last case, authors realized a sensor for DNA sequencing. As a consequence of a target–probe binding event, a conductive bridge forms between the electrodes, resulting in a discrete change of the electrical conductivity with single biorecognition event sensitivity. This tool would be useful in the analysis of mutation in nucleic acid sequence, as it enables the automatic analysis down to two-base mismatches and target amplification techniques (such as PCR) are no longer necessary.

### 5.3. On-Chip Detection of Protein Biomarkers

Once EVs have been lysed, the identification of the proteomic profile of MVs and exosomes has a strong impact in the assessment of the diagnostic and prognostic values of isolated extracellular vesicles. The protein content is typically analyzed by standard techniques, like Western Blot or proteomic methods [226,227], which may led to the identification of novel pathology biomarkers [228,229], but many examples of protein chips based on ELISA-like [230,231,232,233] or other automatized assays [234] may be also listed. As far as on-chip detection of protein biomarkers in exosomes inner content is concerned, this may be conveyed to lab on-chip downstream analytical modules and several efforts were dedicated to improve the limit of detection, including the use of different transduction and signal amplification approaches. 

An optical transduction that is based on fluorescence was recently implemented by Tan and colleagues, who realized a platform that combines photonic crystal enhanced fluorescence (PCEF) detection of a surface-based microspot fluorescent assay with a microfluidic cartridge, in order to achieve pg/mL-level limits of detection for interleukin 3 (IL3) and Tumor Necrosis Factor (TNF-alpha). The fluorescent assay was enhanced 20 times by photonic crystal surface and the whole assay process was completely governed by computer control [235].

Label-free methods can, however, present advantages in terms of assay simplicity and cost. Among them, electrochemical strategies play an important role for lab-on-chip integration providing a good sensitivity and a direct and easily processable electrical signal [236]. Moreover, gold electrodes permit to easily obtain self-assembled layers of capture probes exploiting for example thiol-chemistry [237,238]. 

Innovation however concerned also the capture probes. Recently, Qin and co-workers realized an antibody-free electrochemical biosensor for the selective detection of amyloid-beta oligomers using an electrically conductive poly(pyrrole-2-carboxylic acid) linking agent electropolymerized on the electrode surface and PrPC receptor [239]. They used electrochemical impedance spectroscopy and cyclic voltammetry to fully characterize and measure the analyte binding and they found a very low detection limit of 10^−4^ pM, being very effective for the early diagnosis of Alzheimer’s disease. 

**Figure 12:**
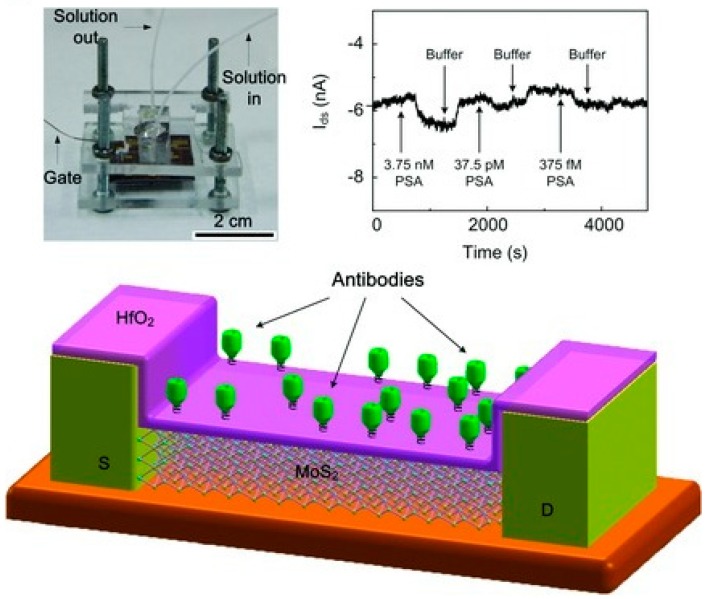
Label-free sensing tool based on molybdenum disulfide (MoS_2_) nanosheet field-effect transistor, in which antibodies against prostate-specific antigens (PSA) are immobilized on the metal surface of the gate, achieving high degrees of sensitivity. Reproduced with permission from reference [243].

Before concluding, it is worth mentioning the opportunities provided by the use of novel nano- and two-dimensional (2D) materials also in this field. Beyond the already broad use of nanoparticles and quantum dots as labels or for signal amplification protocols [240], 2D materials are a relatively new entry in the field driven by the peculiar properties of materials. Research on graphene and related systems (including transition metal dichalcogenides) is opening new perspectives. Graphene-based protein biosensors, for example, have been realized for the highly sensitive detection of biomarkers. The group of Turner and collaborator developed an electrochemical immunosensor that is capable of sensitive and label-free detection of CA 15-3, useful for breast cancer diagnosis. The immunosensor was obtained by modifying a working glassy carbon electrode with antibodies-functionalized N-doped graphene sheets through electrochemical methods [241]. Graphene functionalization methods allowed also for the highly sensitive detection of dopamine onto the surface of a negatively-charged porphyrin film able to attract positively-charged dopamine and to discriminate among dopamine, ascorbic acid, and uric acid, which are known to interfere with dopamine detection [242]. In 2014, Wang and collaborators instead realized a field-effect sensing device based on a molybdenum disulfide (MoS2) multi-layer, demonstrating its ability to detect cancer markers in liquid-phase with a 375 fM limit of detection through a change in the transistor drain current induced by the biorecognition between antibodies immobilized on the MoS2 film surface and prostate-specific antigens (PSA) (Figure 12). 

## 6. Components’ Integration within “Sample-In/Answer-Out” Platforms

One of the major issues in this research topic is the integration of different modules for analysis (separation, counting, characterization) in a single “sample-in/answer-out” platform. 

**Figure 13:**
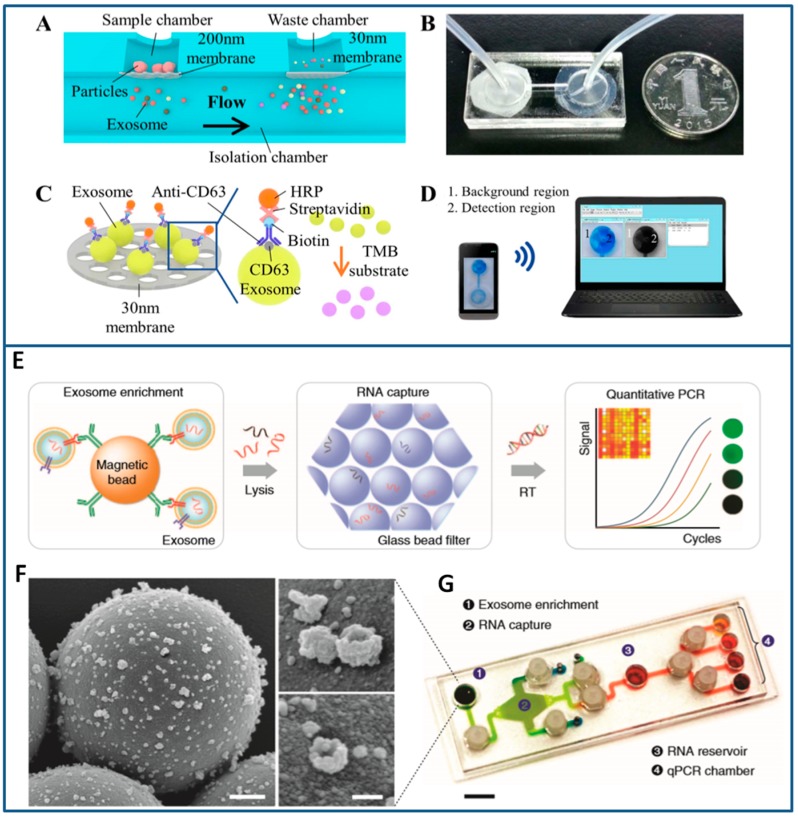
(**A**) Microfluidic platform including a double-filtration module for the size-exclusion for the isolation, enrichment and quantification of exosomes in the isolation chamber (**B**) from urine samples of patients with bladder cancer. Reproduced with permission from reference [143]. The platform allows the labelling of exosomes for an on-chip ELISA and the spots are imaged using a cell phone (**C**,**D**). Another example is the iMER platform that integrates a site for exosomes enrichment through magnetic beads (**E**), a lysis module, a RNA capture module with charged glass beads (**F**) and a qPCR amplification chamber (**G**). Figure reproduced with permission from reference [171].

Some examples of successful integration are already available in literature. For example, in 2017, Liang and co-workers realized a microfluidic platform for the isolation, enrichment, and quantification of exosomes from urine samples of patients with bladder cancer [143]. In their work, they developed a double-filtration based on the principle of size-exclusion. They embedded two polycarbonate membranes with pore sizes ranging from 200 to 30 nm into a PMMA multilayer device, collecting enriched samples of MVs and exosomes in the isolation chamber. The microfluidic system was able to handle large volume of sample (8 mL), which can be continuously flowed through the device and the captured EVs were then analyzed while using an on-chip ELISA in which the development of blue colour was imaged using a cell phone. Images were then transferred via wireless communication to a laptop for image processing (Figure 13A–D).

Another example, even not dealing specifically with EVs, is the work of Lien and collaborators, which were able to develop a compact and automatic tool able to perform leukocytes purification and DNA extraction for fast genetic analysis. The advantages of this device is given by the integration of a microfluidic platform with a bio-separator and a nucleic acid amplification reactor. The optimized platform included circular micropumps, two sets of microcoil arrays, and a membrane type micromixer to mix, transport, and purify the leukocytes samples thanks to CD15/CD45 coated magnetic beads. Subsequent steps allowed for the lysis of cells and the extraction of DNA using surface charge switchable magnetic beads functionalized with specific DNA. The collected DNA was then amplified through an amplification module [204]. 

More recently, Shao and co-workers developed the so-called iMER microfluidic platform, which is able to analyse exosomal mRNA levels of two key enzymes that are involved in glioblastoma multiforme. Levels of the two enzymes in the tumour tissue are monitored to evaluate drug treatment efficacy. The developed microfluidic platform provided the enrichment of cancer-specific exosomes through immunomagnetic selection from blood samples, then they were lysed on the same chip and their RNA content was captured through glass beads and subsequently processed by real-time PCR [171] (Figure 13E–G). 

To support all the steps of sample preparation, analysis and waste, the realization of a compact testing platform should take into consideration that microfluidics usually incorporate different materials and fabrication techniques, as well as many different functional elements. Increasing chip complexity adds redundancy to the interconnection between different modules and often it is easier to renounce to some degree of automation and fall back down to simpler devices. For example, microfluidic devices usually have to include many buffer reservoirs for the automatic delivery of reactions solutions and an efficient sealing of fluidic connections among them and with electronic systems, is still a bottleneck of integration at scalable dimensions, making the bonding techniques and materials used in the package more important than ever [244].

## 7. Conclusions

Role and analysis of extracellular vesicles, including microvesicles and exosomes, are of undoubted scientific interest for diagnostic and prognostic value. However, despite their promising utility as biomarkers for various diseases, their use in clinical settings is still far from everyday practice due to the high difficulty in their detection and characterization using standard techniques of analysis. In this review, we discussed their clinical value and limitations of current methods for EVs analysis and we focused on innovative approaches that are able to push a significant progress toward a better exploitation of the clinical potential of extracellular vesicles. In particular, this work encompasses the panorama of methods and technologies available or applicable in the field of lab-on-chip dealing with exosomes, including modules for sorting and concentration of exosomes (both passive and active), for on-chip investigation of their content, which is expected to reveal a new frontier in targeting the early diagnosis of several pathologies. The integration of single modules for analysis of EVs into complex lab-on-chips is also one of the targets to be achieved in order to make technology closer to the clinical practice and thus to be exploited at the industrial level and to approach clinical practice and the market. Progresses in micro- and nanotechnologies, together with recent improvements in the exploitation of new and smart materials, have the power and possibility to boost these innovations into reality, leading to a turning point in standard medicine. 

Overcoming the gap between high specialized research and the concrete use of developed tools in common settings is indeed the other big challenge for research dealing with miniaturization and point-of-care devices. In conclusion, the combination of EVs analysis and lab-on-chip technologies could be expected to pave the way to a new era in precision medicine and personalized therapies.

## Figures and Tables

**Table 1:** Methods for Extracellular Vesicles (EV) isolation.

Isolation Method	Isolation Principle	Advantages/Limitations
Differential centrifugation	EV separation based on particle density, size and shape	- Commonly used; standardized; vesicle enrichment as pellet; EV subtypes isolation by density gradient centrifugation - Vesicle aggregation; protein and soluble factors contamination; low recovery; laborious
Polymer-based precipitation	EV precipitation using polymers altering solubility	- Easy and inexpensive; high yield; effective with small amount of starting material; preservation of bioactivity - Co-precipitation of protein contaminants and polymeric materials; not suitable for large scale studies; long incubation times
Size-exclusion chromatography (SEC)	EV isolation by gel filtration chromatography based on size	- Inexpensive; reproducible; high yield and purity; preservation of integrity and activity. - Specific equipment; not suitable for large scale studies; long run times.
Immunoaffinity capture-based techniques	EV immuno-purification using magnetic beads conjugated with antibodies direct toward specific EV surface markers	- Sensitivity; specificity; high purity; EV subtypes isolation. - Expensive; antibody cross-reactivity; low yield

**Table 2:** Methods for EV characterization.

Method	Information Acquired	Advantages/Limitations
Electron microscopy (EM)	EV dimension and morphology	- Direct assessment of morphology and size; small sample amount - Time consuming; size and morphology modifications due to sample preparation
Atomic force microscopy (AFM)	EV three-dimensional topography	- No sample fixation and staining; small sample amount - Size and morphology modifications due to sample dehydration on mica surface
Dynamic light scattering (DLS)	EV size distribution	- Fast; no sample preparation; sample preservation for downstream analysis - Inaccurate with polydispersed and size heterogeneous samples
Nanoparticle tracking analysis (NTA)	EV concentration and size distribution	- Fast; no sample preparation; sample preservation for downstream analysis - Inaccurate with size heterogeneous samples and particle aggregates
Tunable resistive pulse sensing (TRPS)	EV concentration, size distribution and surface charge	- Fast; no sample preparation - Difficulties with unknown and heterogeneous size distribution samples (difficult to select the correct nanopore setup); detection of non-vesicular material within size range
Flow cytometry	EV marker characterization, absolute counting	- Quantitative and qualitative (using specific antibodies) characterization of EVs - Detection limit (>100 nm, flow cytometer dependent); swarming effect (identification of multiple vesicles as a single event); detection of protein/antibody aggregates
ELISA/Western Blot	EV protein quantification	- Standard immunological methods; specific characterization of EV protein markers - Time consuming; possible detection of non-EV proteins; non-specific information on EV concentration/size/distribution

**Table 3:** Summary of passive methods to isolate EVs, with schemes of working of the main techniques. (A) immunoaffinity methods include the functionalization of the channel with antibodies able to hold the desired microvesicles (MVs), (B) size-exclusion scheme for the entrapment of exosomes (reprinted with permission of [143]), (C) working principle of flow-induced method (reprinted with permission from reference [152], Copyright 2017 American Chemical Society).

Separation Method	Advantages/Limitations	Size Range/Sorted Items:	Technical Details
**Immunoaffinity** 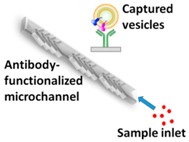 (A)	- High purity of the isolated exosomes (specific separation) - Simple chip structure (low complexity in fabrication) - Fast separation - Only exosomes with targeted proteins can be separated - Needs of strategies to reduce non-specific exosome adsorption and enhance mixing	**~20–135 nm [133]; 30–300 nm [112]** - exosomes from serum [112,133] **<150 nm** - exosomes from plasma [135] **~20–260 nm** - exosomes from ascites [136] **~30–120 nm** - exosome from cell culture medium through polystyrene beads [114]	**Sample Amount:** 20–400 µL [115,133,135,136] **Sample Pre-treatment:** filtration [133,136] dilution [135], RBC lysis and pre-incubation [115] **Recovery yields:** 42–94% [133] **Isolation throughput:** 0.05–13.1 µL/min [115,133,135,136]; 70 µL/min [114]
**Size-exclusion** 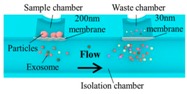 (B)	- Size uniformity of the separated exosomes - No contamination of non-exosomial proteins or microvesicles - High risk of chip clogging for processing of large amount of sample, saturation limits, - Complicated fabrication processes	**~150 nm** - exosomes from whole blood [141,146] **~170 nm** - erythrocyte-derived microvesiscles [146] **30–200 nm [143,146]; 20−600 nm [144]**—exosomes from urine [143,144,146] **20−600 nm [144]** - exosomes from cell-culture supernatant [144] **~83–120 nm** - model samples like liposomes [145]	**Sample Amount:** 4–300 µL [141,142,145]; 1–8 mL [144,146] **Sample Pre-treatment:** filtration [143]; centrifugation [143,146] **Recovery yields:** >3% [141]; 60–95% [143,144,145] **Isolation throughput:** 0.075–3 µL/min [141,142,146]; 10–36 µL/min [143,144,145]
**Flow-induced** 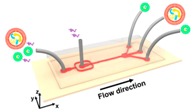 (C)	- Very low amount of sample - High purity and size controlled recovery of exosomes -Simple microchannel structures (except DLD) - Long processing times (in DLD) - High clogging risks (in DLD) - Complex design evaluation - Skill for operations	**<100 nm** - exosomes from cell culture medium [112] and commercial urine [113] (***DLD***) **30–100 nm** - exosome from cells culture media [150] (***PFF***) **<200 nm** -exosomes from FBS [152] (***Viscoelastic Microfluidics***)	**Sample Amount:** 0.72 µL [113]; 100 µL [152] **Recovery yields:** 39% [112]; 93.6% [152] **Recovery purity:** >90% [112,152] **Isolation throughput:** 0.1–0.2 nL/min [113]; 3–10 µL/min [150,152]

**Table 4:** Summary of active methods to isolate EVs, with schemes of working of the main techniques. (**A**) Scheme of dielectrophoretic separation of particles reproduced from reference [185]; (**B**) Magnetic separation of captured analytes; and, (**C**) Sorting scheme for Surface acoustic waves separation of particles, reproduced with permission from reference [181].

Separation Method	Advantages/Limitations	Size Range/Sorted Items	Technical Details
**Electroactive** **(*Dielectrophoresis, Electro-osmosis*)** 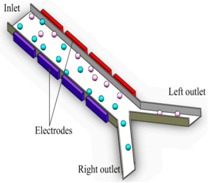 (A)	- Suitability for miniaturization of active components and integration into microfluidic devices - No need for antibodies - Viability of cells at the end of the separation process - heavy benchtop instrumentation for the generation of electric fields - Possible heating of solution - Water electrolysis	**~50–400 nm** **≤10 μm in case of blood cells** - micro/nanoparticles from solution [159,166] *-* exosomes [165,166] and cells [162] from blood (***Dielectrophoresis***) - particles and cells from solution [186] (***Electro-osmosis***)	**Sample amount:** 0.4 μL–25 μL [159,165] at 1 mL/h [159] **Sample pre-treatment:** undiluted plasma [165]; 2 μL of blood mixed with 2 μL heparine sodium [162] **Isolation throughput:** High selectivity: - Particles from liposomes separation [166]; up to 90% for one type of particle [159] - 1 μm particles concentrated applying 100–200 V ac field [186]; - 4.6 particles/s Applying a potential of 10 V/cm [166]
**Magnetic** 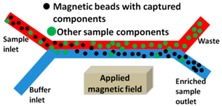 (B)	- No damages on cells - No heating of solution - No influence from pH or ionic strength - Suitable for miniaturization and integration into microfluidic devices - Usually it needs bio-functionalized beads (antibody-based isolation)	**~30–100 nm/0.5–5 μm for beads** - exosomes from plasma [187] or serum [171]	**Sample amount:** - 500 μL of beads suspension flowed at 100 μL/min [168] - 20 μL–100 μL of serum/plasma [172,188] **Isolation throughput:** recovery efficiency of 72% at 1 μL/min [172]
**Acoustic** 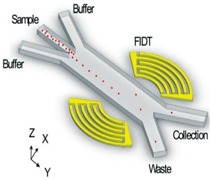 (C)	- No damages on cells - No heating of solution - No influence from pH or ionic strength - No need for antibodies - Suitable for miniaturization of active components - heavy benchtop instrumentation needed for the generation of acoustic waves	**~30 nm–10 μm** - particles [116] from solution - cells and circulating tumour cells [182] from blood - exosomes [184] from serum	**Sample amount:** - 0.3 μL/min [182]; **Sample preparation:** - Whole blood with PBS with/without dextrane as sheat flow [182,184] **Isolation throughput:** - Cell removal module efficiency: 99% of 5 μm particles and exosomes recovery at 8 μL/min [184] ~90% of 5 μm particles [182]
